# Functional Characterization of Putative Novel MicroRNA-1133 in the Antioxidant Effects of Metformin in Hyperglycemia-Induced Endothelial Cells

**DOI:** 10.3390/ijms27177556

**Published:** 2026-08-24

**Authors:** Nur Syakirah Othman, Amilia Aminuddin, Adila A. Hamid, Shahidee Zainal Abidin, Saiful Effendi Syafruddin, Mohd Faizal Ahmad, Farah Hanan Fathihah Jaffar, Nur Athirah Othman Basri, Azizah Ugusman

**Affiliations:** 1 Department of Physiology, Faculty of Medicine, Universiti Kebangsaan Malaysia, Kuala Lumpur 56000, Malaysia; dr.nursyakirahothman@gmail.com (N.S.O.); amilia@hctm.ukm.edu.my (A.A.); adilahamid@ukm.edu.my (A.A.H.); farahhanan@ukm.edu.my (F.H.F.J.); p131665@siswa.ukm.edu.my (N.A.O.B.); 2 Cardiovascular and Pulmonary (CardioResp) Research Group, Universiti Kebangsaan Malaysia, Bangi 43600, Malaysia; 3 Faculty of Science and Marine Environment, Universiti Malaysia Terengganu, Kuala Nerus 21030, Malaysia; shahidee.zainal@umt.edu.my; 4 UKM Medical Molecular Biology Institute (UMBI), Universiti Kebangsaan Malaysia, Kuala Lumpur 56000, Malaysia; effendisy@hctm.ukm.edu.my; 5 Department of Obstetrics and Gynecology, Faculty of Medicine, Universiti Kebangsaan Malaysia, Kuala Lumpur 56000, Malaysia; drmohdfaizal@ukm.edu.my; 6 Faculty of Health Sciences, MAIWP International University, Kuala Lumpur 68100, Malaysia

**Keywords:** diabetes mellitus, endothelial dysfunction, hyperglycemia, metformin, novel miR-1133, oxidative stress, PIK3CA

## Abstract

Diabetes mellitus induces endothelial dysfunction and oxidative stress, contributing to the development of vascular complications. Although metformin exerts well-established vasculoprotective and antioxidant effects, the molecular mechanisms underlying these actions remain incompletely understood. Novel miR-1133 was previously identified as a metformin-responsive putative microRNA (miRNA), and bioinformatic analysis identified PIK3CA as a biologically plausible candidate target for further investigation. This study investigated the functional significance of novel miR-1133 in the antioxidant effects of metformin in hyperglycemia-induced human umbilical vein endothelial cells (HUVECs). HUVECs were exposed to normal glucose (5.5 mmol/L), hyperglycemia (33.3 mmol/L), or hyperglycemia supplemented with metformin (10 μM) for 24 h. Metformin-treated hyperglycemic HUVECs were subsequently transfected with a novel miR-1133 mimic during continued hyperglycemic and metformin exposure. PIK3CA expression and oxidative stress markers, including reactive oxygen species (ROS), superoxide dismutase (SOD), and 8-hydroxy-2′-deoxyguanosine (8-OHdG), were evaluated. Hyperglycemia increased novel miR-1133 expression, whereas metformin reduced its expression. Metformin treatment was associated with increased PIK3CA mRNA and protein expression, reduced ROS and 8-OHdG levels, and increased SOD expression and activity. Novel miR-1133 mimic transfection was associated with reduced PIK3CA expression, increased ROS and 8-OHdG levels, and decreased SOD expression and activity compared with the corresponding transfection control. Collectively, these findings demonstrate that metformin attenuates oxidative stress in hyperglycemia-induced endothelial cells and overall provide the first functional characterization of novel miR-1133. The reciprocal changes in PIK3CA expression and oxidative stress observed following novel miR-1133 mimic transfection suggest that novel miR-1133 may represent a candidate mediator of the endothelial antioxidant responses associated with metformin. Further studies are required to validate the direct interaction between novel miR-1133 and PIK3CA and to elucidate the underlying molecular mechanisms.

## 1. Introduction

Diabetes mellitus (DM) is a major global health problem, affecting more than 800 million individuals worldwide [1]. Chronic hyperglycemia is a hallmark of DM and contributes significantly to the development of vascular complications, which remain the leading cause of morbidity and mortality among diabetic patients. Endothelial dysfunction is a key early event in diabetic vascular disease and is largely driven by excessive oxidative stress resulting from prolonged exposure to hyperglycemia [2].

Hyperglycemia promotes excessive production of reactive oxygen species (ROS), resulting in oxidative damage to proteins, lipids, and nucleic acids. Excessive ROS disrupts endothelial homeostasis by reducing nitric oxide bioavailability while promoting inflammation and apoptosis, thereby contributing to vascular dysfunction [3]. Oxidative DNA damage can be assessed by measuring 8-hydroxy-2′-deoxyguanosine (8-OHdG), a well-established biomarker of oxidative stress [4]. Under physiological conditions, endogenous antioxidant enzymes, including superoxide dismutase (SOD), catalase, and glutathione peroxidase, protect endothelial cells against oxidative injury. However, these antioxidant defense systems become impaired under persistent hyperglycemic conditions, further exacerbating endothelial dysfunction [5].

MicroRNAs (miRNAs) are small non-coding RNAs that regulate gene expression at the post-transcriptional level and play important roles in endothelial homeostasis. Increasing evidence indicates that dysregulated miRNA expression contributes to diabetic vascular complications by modulating oxidative stress, inflammation, apoptosis, and endothelial dysfunction [6]. Furthermore, accumulating evidence suggests that part of the therapeutic effects of several pharmacological agents may be mediated through modulation of miRNA expression [7]. Among these agents, metformin has been shown to influence the expression of circulating miRNAs in patients with type 2 DM, suggesting that miRNA-mediated regulation may contribute to its vasculoprotective actions [8].

Metformin is the first-line pharmacological therapy for type 2 DM and has been shown to provide vascular protective effects beyond glycemic control [9]. Previous studies have demonstrated that metformin attenuates oxidative stress, improves endothelial function, and protects against vascular injury through multiple signaling pathways involved in cellular survival, metabolism, and antioxidant defense [10,11]. Despite these established vasculoprotective effects, the contribution of individual miRNAs to the antioxidant mechanisms of metformin remains incompletely understood, particularly those that have not yet been functionally characterized. While numerous studies have focused on annotated miRNAs, advances in next-generation sequencing continue to identify previously uncharacterized miRNAs that may also contribute to disease pathogenesis and therapeutic responses. The functional significance of these putative novel miRNAs, however, remains largely unexplored.

In our previous small RNA sequencing study, which examined the effects of hyperglycemia on human umbilical vein endothelial cells (HUVECs), we identified a putative novel miRNA designated novel miR-1133 [12]. This candidate miRNA was predicted using the miRDeep2 algorithm following alignment of sequencing reads to the human reference genome and comparison with known miRNAs in miRBase and was subsequently validated by stem-loop RT-qPCR [12]. At the time of the study, novel miR-1133 had not been annotated in public miRNA repositories, and its biological function remained unknown.

Our previous study also demonstrated that metformin significantly altered the miRNA expression profile of hyperglycemia-induced HUVECs, with novel miR-1133 identified as one of the most significantly downregulated miRNAs following metformin treatment [13]. Subsequent bioinformatic analyses demonstrated that novel miR-1133 possessed the largest number of predicted target genes among the downregulated miRNAs and that its predicted target genes were enriched in several diabetes-related pathways, including the phosphatidylinositol signaling pathway [13]. Furthermore, protein–protein interaction network analysis identified phosphatidylinositol-4,5-bisphosphate 3-kinase catalytic subunit alpha (PIK3CA) as one of the hub genes within the predicted regulatory network, suggesting its potential biological relevance in metformin-mediated endothelial protection [13]. Given the established role of phosphatidylinositol 3-kinase (PI3K) signaling in endothelial survival, redox homeostasis, and vascular function [14,15,16,17,18], PIK3CA was selected as a biologically plausible candidate for further investigation. However, although these bioinformatic findings generated a plausible mechanistic hypothesis, the functional significance of novel miR-1133 in the antioxidant effects of metformin and its association with PIK3CA signaling have not yet been experimentally investigated.

Therefore, the present study investigated the functional characterization of novel miR-1133 in metformin-treated hyperglycemic endothelial cells. Specifically, we examined whether experimental modulation of novel miR-1133 using a synthetic miRNA mimic was associated with changes in PIK3CA expression and oxidative stress markers, including ROS production, SOD expression and activity, and 8-OHdG levels. By providing the first functional characterization of novel miR-1133, this study sought to determine whether modulation of this previously uncharacterized putative miRNA is associated with the antioxidant effects of metformin in hyperglycemia-induced endothelial cells and to generate a foundation for future mechanistic investigations.

## 2. Results

### 2.1. Metformin Reduced Novel miR-1133 Expression in Hyperglycemia-Induced HUVECs

**Figure 1:**
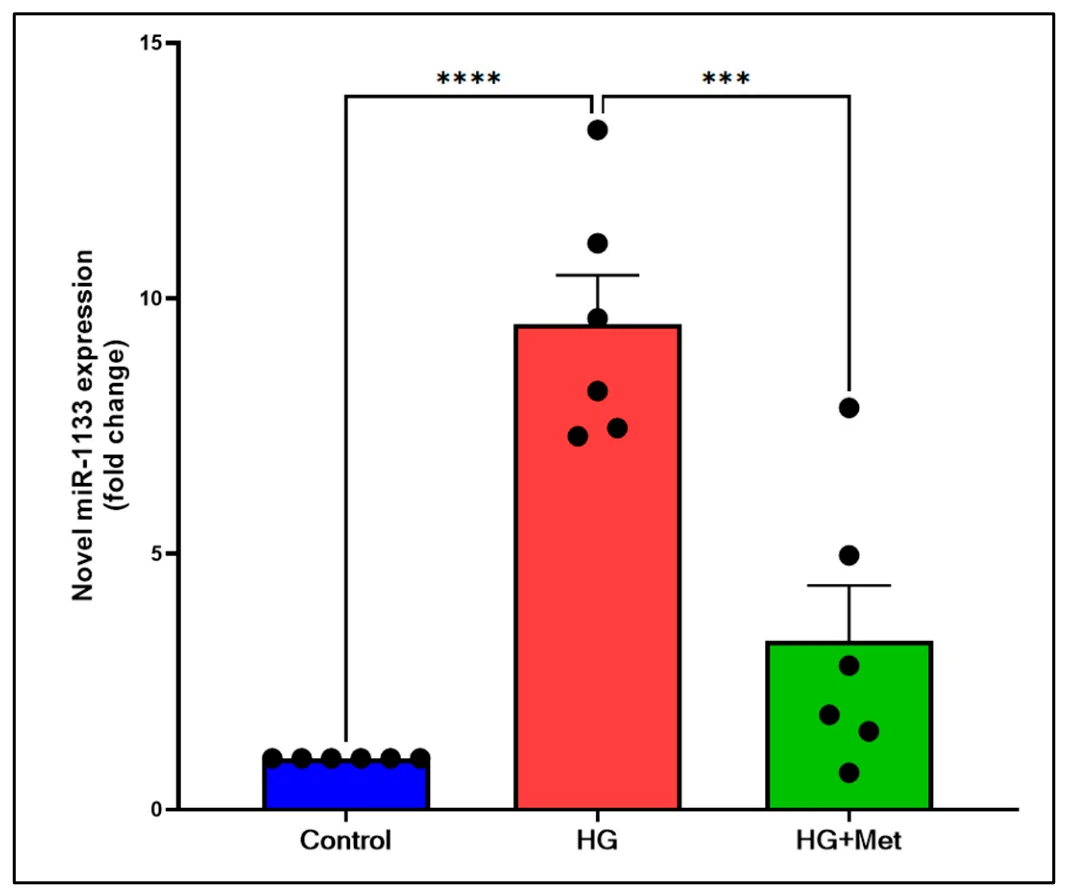
Expression of novel miR-1133 in HUVECs exposed to normal glucose (Control), hyperglycemia (HG) and hyperglycemia with metformin (Met) treatment (HG + Met). Data are presented as mean ± SEM (*n* = 6); *** *p* < 0.001, **** *p* < 0.0001.

Stem-loop RT-qPCR analysis demonstrated that novel miR-1133 expression was significantly higher in hyperglycemia (HG)-induced HUVECs than in the control group (*p* < 0.0001). Treatment with metformin significantly reduced novel miR-1133 expression compared with the HG group (*p* < 0.001) (Figure 1). These findings indicate that hyperglycemia was associated with increased novel miR-1133 expression, whereas metformin treatment was associated with a reduction in novel miR-1133 expression.

### 2.2. Novel miR-1133 Mimic Transfection Is Associated with Reduced PIK3CA Expression in Metformin-Treated Hyperglycemia-Induced HUVECs

**Figure 2:**
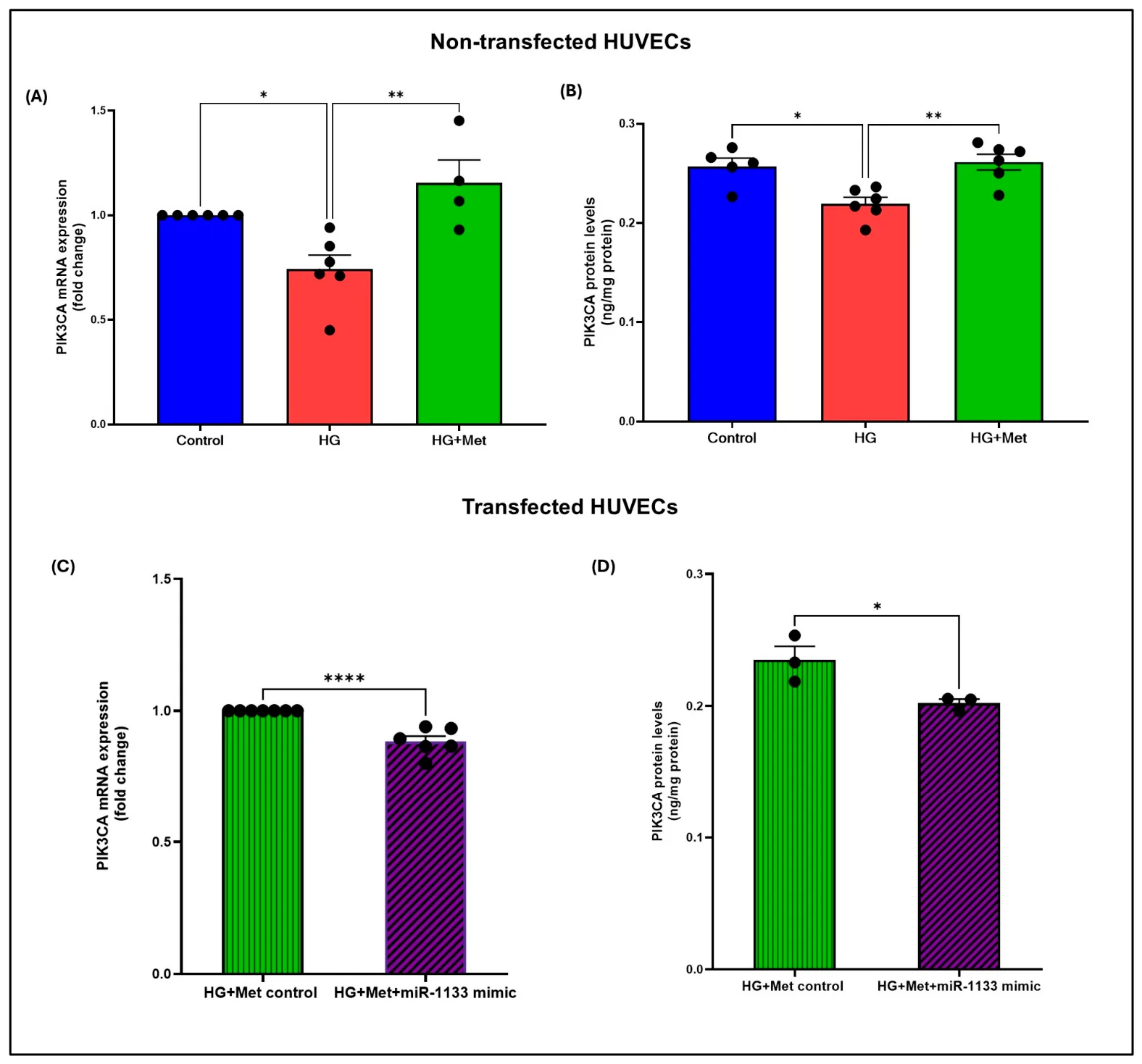
PIK3CA mRNA and protein expression in hyperglycemia-induced HUVECs following metformin treatment and novel miR-1133 mimic transfection. (**A**) PIK3CA mRNA expression in non-transfected HUVECs, (**B**) PIK3CA protein levels in non-transfected HUVECs, (**C**) PIK3CA mRNA expression in HG + Met-treated HUVECs following novel miR-1133 mimic transfection, and (**D**) PIK3CA protein levels in HG + Met-treated HUVECs following novel miR-1133 mimic transfection. The transfection control group consisted of HG + Met-treated HUVECs transfected with Lipofectamine^®^ only. Data are presented as mean ± SEM (*n* = 3–6); * *p* < 0.05, ** *p* < 0.01 and **** *p* < 0.0001. HG, hyperglycemia; HUVECs, human umbilical vein endothelial cells; Met, metformin; mRNA, messenger RNA; PIK3CA, phosphatidylinositol-4,5-bisphosphate 3-kinase catalytic subunit alpha.

Treatment of HG-induced HUVECs with metformin significantly increased PIK3CA mRNA expression compared with the HG group (*p* < 0.05) (Figure 2A). Similarly, PIK3CA protein levels were significantly elevated following metformin treatment compared with the HG group (*p* < 0.01) (Figure 2B). To investigate the functional significance of novel miR-1133, HG + Met-treated HUVECs were transfected with a novel miR-1133 mimic during continued hyperglycemic and metformin exposure. Novel miR-1133 mimic transfection was associated with a significant reduction in PIK3CA mRNA expression compared with the transfection control group (*p* < 0.0001) (Figure 2C). Consistent with the mRNA findings, PIK3CA protein levels were also significantly lower following novel miR-1133 mimic transfection (*p* < 0.05) (Figure 2D). Collectively, these findings demonstrate that novel miR-1133 mimic transfection was associated with reduced PIK3CA mRNA and protein expression in metformin-treated hyperglycemic HUVECs.

### 2.3. Novel miR-1133 Mimic Transfection Is Associated with Increased Oxidative Stress in Metformin-Treated Hyperglycemia-Induced HUVECs

#### 2.3.1. Intracellular ROS Levels

**Figure 3:**
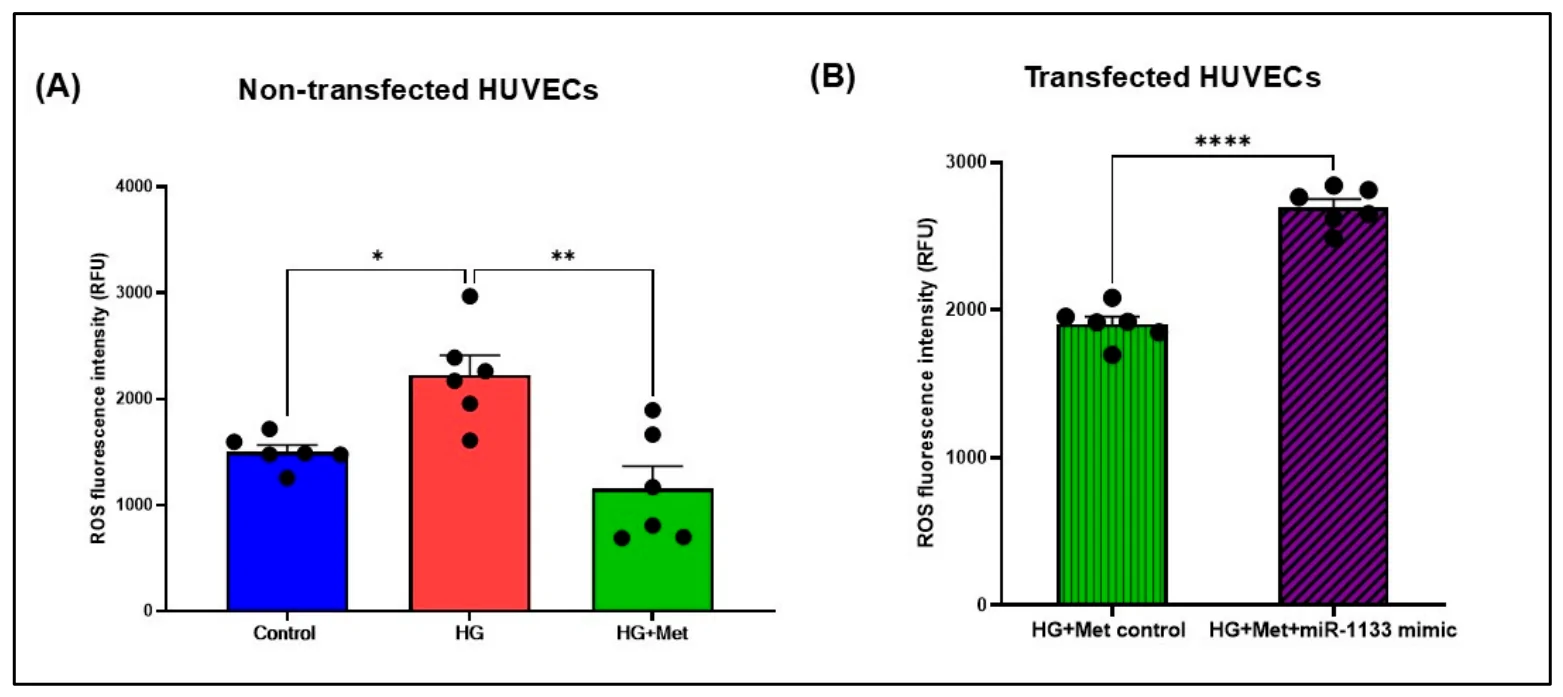
Intracellular reactive oxygen species (ROS) levels in hyperglycemia (HG)-induced human umbilical vein endothelial cells (HUVECs) treated with metformin (Met). (**A**) ROS levels in non-transfected HUVECs and (**B**) ROS levels in HG + Met-treated HUVECs following novel miR-1133 mimic transfection. The transfection control group consisted of HG + Met-treated HUVECs transfected with Lipofectamine^®^ only. Data are presented as mean ± SEM (*n* = 6). * *p* < 0.05, ** *p* < 0.01, **** *p* < 0.0001.

Hyperglycemia significantly increased intracellular ROS levels compared with the control group (*p* < 0.05), whereas metformin treatment significantly reduced ROS production (*p* < 0.01) (Figure 3A). Novel miR-1133 mimic transfection was associated with significantly higher intracellular ROS levels than in the transfection control group (*p* < 0.0001) (Figure 3B).

#### 2.3.2. SOD1 mRNA Expression

**Figure 4:**
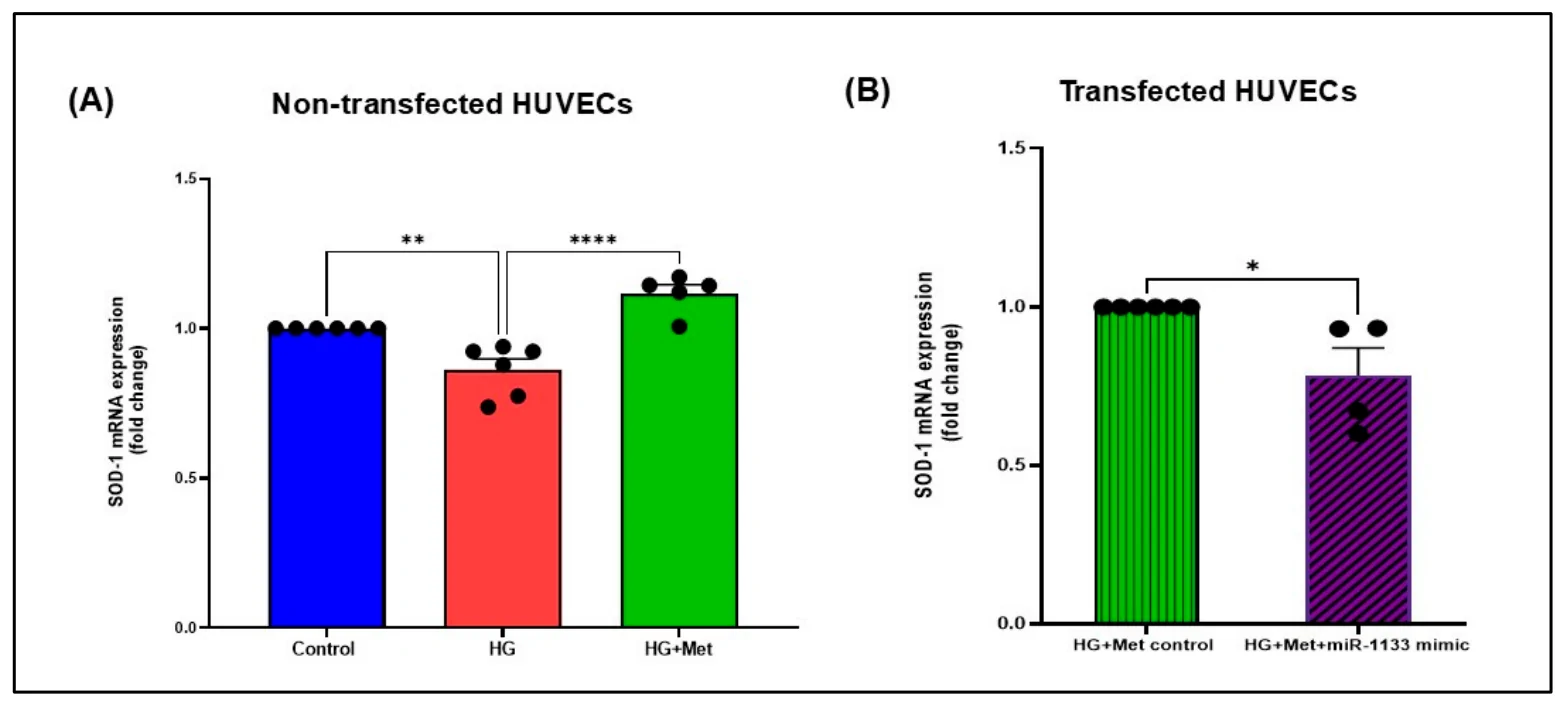
Superoxide dismutase-1 (SOD1) mRNA expression in hyperglycemia (HG)-induced human umbilical vein endothelial cells (HUVECs) treated with metformin (Met). (**A**) SOD1 mRNA expression in non-transfected HUVECs and (**B**) SOD1 mRNA expression in HG + Met-treated HUVECs following novel miR-1133 mimic transfection. The transfection control group consisted of HG + Met-treated HUVECs transfected with Lipofectamine^®^ only. Data are presented as mean ± SEM (*n* = 4–6); * *p* < 0.05, ** *p* < 0.01, **** *p* < 0.0001.

Hyperglycemia significantly reduced SOD1 mRNA expression compared with the control group (*p* < 0.01). Treatment with metformin significantly increased SOD1 mRNA expression compared with the HG group (*p* < 0.0001) (Figure 4A). Novel miR-1133 mimic transfection was associated with significantly lower SOD1 mRNA expression than in the transfection control group (*p* < 0.05) (Figure 4B).

#### 2.3.3. Total SOD Activity

**Figure 5:**
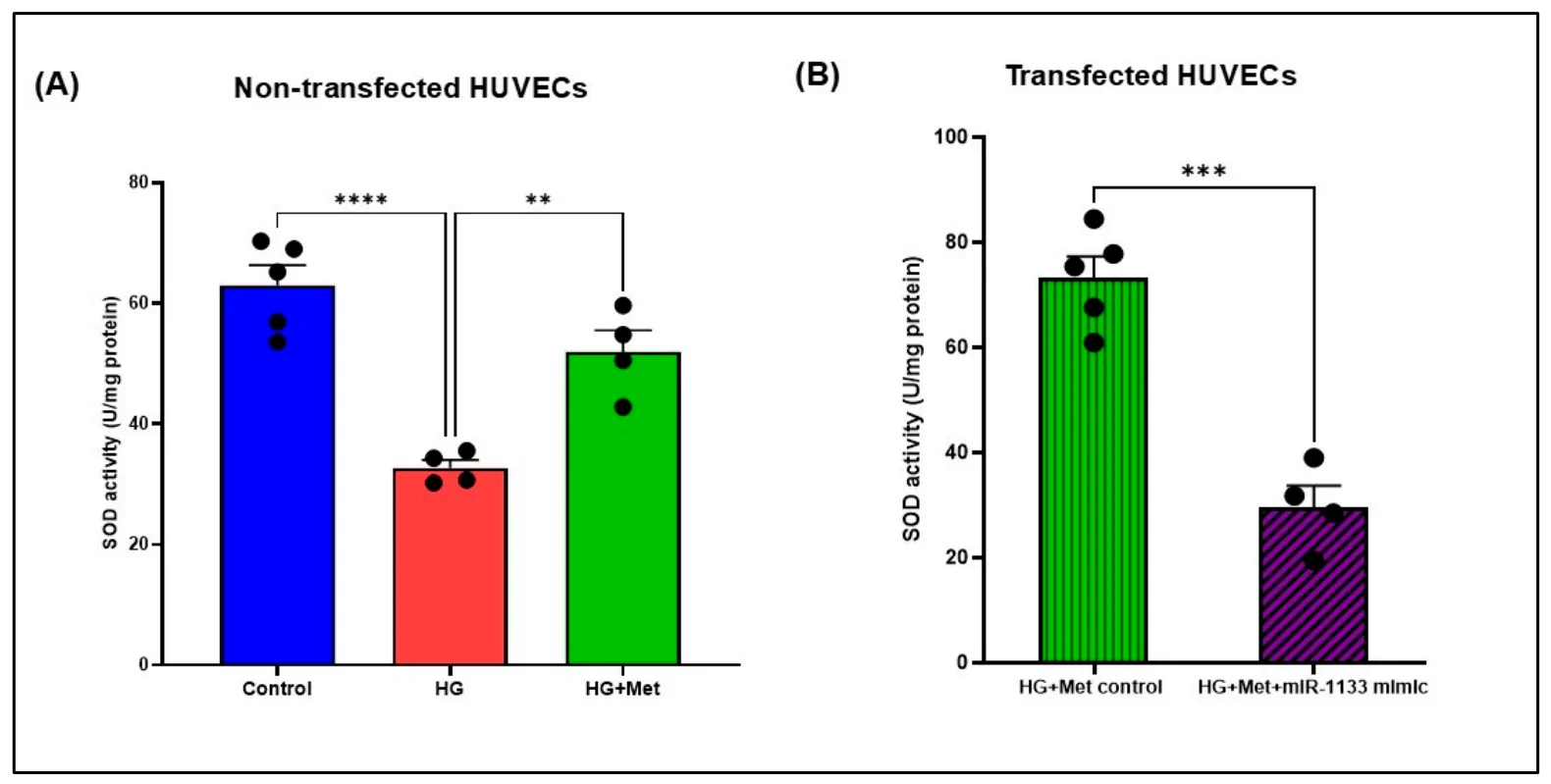
Total superoxide dismutase (SOD) activity in hyperglycemia (HG)-induced human umbilical vein endothelial cells (HUVECs) treated with metformin (Met). (**A**) SOD activity in non-transfected HUVECs and (**B**) SOD activity in HG + Met-treated HUVECs following novel miR-1133 mimic transfection. The transfection control group consisted of HG + Met-treated HUVECs transfected with Lipofectamine^®^ only. Data are presented as mean ± SEM (*n* = 4–5); ** *p* < 0.01, *** *p* < 0.001, **** *p* < 0.0001.

Total SOD activity was significantly reduced in HG-induced HUVECs compared with the control group (*p* < 0.0001), whereas metformin treatment significantly increased SOD activity compared with the HG group (*p* < 0.01) (Figure 5A). Novel miR-1133 mimic transfection was associated with significantly lower SOD activity than in the transfection control group (*p* < 0.001) (Figure 5B).

#### 2.3.4. 8-OHdG Levels

**Figure 6:**
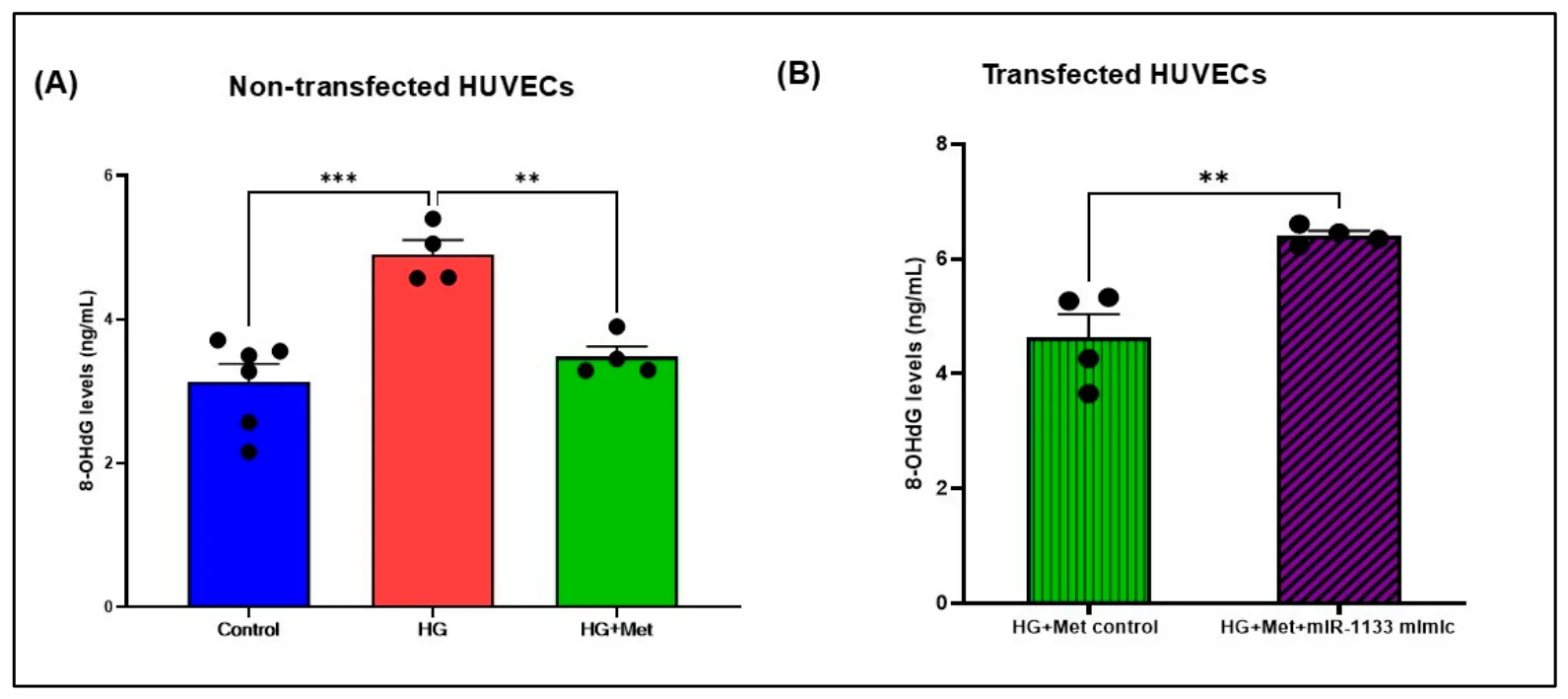
The levels of 8-hydroxy-2′-deoxyguanosine (8-OHdG) in hyperglycemia (HG)-induced human umbilical vein endothelial cells (HUVECs) treated with metformin (Met). (**A**) 8-OHdG levels in non-transfected HUVECs and (**B**) 8-OHdG levels in HG + Met-treated HUVECs following novel miR-1133 mimic transfection. The transfection control group consisted of HG + Met-treated HUVECs transfected with Lipofectamine^®^ only. Data are presented as mean ± SEM (*n* = 4–6); ** *p* < 0.01, *** *p* < 0.001.

Hyperglycemia significantly increased 8-OHdG levels compared with the control group (*p* < 0.001), whereas metformin treatment significantly reduced 8-OHdG levels (*p* < 0.01) (Figure 6A). Novel miR-1133 mimic transfection was associated with significantly higher 8-OHdG levels than in the transfection control group (*p* < 0.01) (Figure 6B).

## 3. Discussion

The present study provides the first functional characterization of novel miR-1133 in hyperglycemia-induced endothelial cells treated with metformin. The findings demonstrated that hyperglycemia significantly increased novel miR-1133 expression, whereas metformin treatment reduced its expression. These changes were associated with increased PIK3CA mRNA and protein expression, decreased intracellular ROS and 8-OHdG levels, and increased SOD1 expression and total SOD activity. In the transfection experiment, introduction of the novel miR-1133 mimic during continued HG + Met treatment was associated with reduced PIK3CA expression, increased oxidative stress, and attenuation of the antioxidant responses observed with metformin. Collectively, these findings suggest that modulation of novel miR-1133 is associated with reciprocal changes in PIK3CA expression and oxidative stress during metformin treatment under hyperglycemic conditions. However, these observations should be interpreted as evidence of a functional association rather than proof of direct molecular regulation.

Novel miR-1133 is a putative novel miRNA previously identified using the miRDeep2 prediction algorithm and subsequently validated by stem-loop RT-qPCR [12]. Further independent annotation and deposition in public miRNA databases will nevertheless be required before it can be established as a bona fide human miRNA. In our previous small RNA sequencing study, novel miR-1133 was among the most significantly downregulated novel miRNAs following metformin treatment in hyperglycemia-induced HUVECs [13]. Bioinformatic analysis further showed that it had the largest number of predicted target genes among the downregulated novel miRNAs, with enrichment of several pathways relevant to endothelial function, including phosphatidylinositol signaling. Protein–protein interaction network analysis also identified PIK3CA as a hub gene within the predicted regulatory network [13]. These observations provided the rationale for examining the relationship between novel miR-1133, PIK3CA expression, and oxidative stress under the same experimental conditions in the present study. Although the biological function of novel miR-1133 remains incompletely characterized, the current findings suggest that it may represent a candidate mediator of the endothelial response to metformin during hyperglycemic stress.

An important finding of the present study was that metformin treatment was associated with increased PIK3CA mRNA and protein expression in hyperglycemia-induced HUVECs. PIK3CA encodes the p110α catalytic subunit of PI3K, which plays a central role in regulating endothelial cell survival, metabolism, and redox homeostasis through activation of the PI3K/Akt signaling pathway [14,19]. Previous studies have demonstrated that activation of PI3K/Akt signaling protects endothelial cells against hyperglycemia-induced injury by enhancing antioxidant defense mechanisms, promoting cell survival, and reducing oxidative stress [14,15,16,17,18]. Furthermore, metformin has been reported to activate PI3K/Akt signaling, either directly or through crosstalk with other signaling pathways such as AMPK, thereby contributing to improved endothelial function and vascular protection [20]. Therefore, the increased PIK3CA expression observed following metformin treatment in the present study is consistent with the established vasculoprotective actions of metformin.

To further explore the functional significance of novel miR-1133, HG + Met-treated HUVECs were transfected with a novel miR-1133 mimic during continued hyperglycemic and metformin exposure. Mimic transfection was associated with significant reductions in both PIK3CA mRNA and protein expression compared with the corresponding Lipofectamine^®^-only transfection control. Our previous RNA sequencing study identified novel miR-1133 as a putative metformin-responsive miRNA [13]. The present study builds upon those findings by providing the first functional characterization of novel miR-1133 and demonstrating that its experimental modulation is associated with reciprocal changes in PIK3CA expression. However, the present study did not directly determine whether PIK3CA is a direct molecular target of novel miR-1133. Consequently, the observed inverse relationship should be interpreted as evidence of a functional association rather than definitive proof of direct post-transcriptional regulation. Validation using dual-luciferase reporter assays or other target engagement approaches will be required to establish whether PIK3CA is directly regulated by novel miR-1133.

Beyond its association with PIK3CA expression, modulation of novel miR-1133 was accompanied by marked changes in cellular oxidative stress. Hyperglycemia significantly increased intracellular ROS production, whereas metformin reduced ROS levels, consistent with numerous studies demonstrating the antioxidant properties of metformin in endothelial cells [21,22,23,24]. These antioxidant effects have been attributed to multiple mechanisms, including inhibition of NADPH oxidase activity, activation of AMPK signaling, and suppression of mitochondrial ROS generation through inhibition of respiratory chain complex I [21,22,23,24]. Novel miR-1133 mimic transfection during continued HG + Met treatment was associated with a significant increase in intracellular ROS compared with the Lipofectamine^®^-only control, suggesting that elevated novel miR-1133 expression may attenuate the antioxidant responses associated with metformin under hyperglycemic conditions.

A similar pattern was observed in the endogenous antioxidant defense system. Metformin significantly increased both SOD1 mRNA expression and total SOD activity, whereas novel miR-1133 mimic transfection was associated with reductions in both parameters. These findings are consistent with previous reports demonstrating that metformin enhances endogenous antioxidant defenses by upregulating antioxidant enzymes, including SOD, catalase, and glutathione peroxidase [25,26]. As SOD represents one of the principal enzymatic defenses against superoxide radicals, the reduction in SOD expression and activity observed following novel miR-1133 mimic transfection may have contributed to the accompanying increase in intracellular ROS.

Consistent with the observed changes in intracellular ROS, metformin significantly reduced 8-OHdG levels, indicating attenuation of oxidative DNA damage under hyperglycemic conditions. These findings agree with previous reports demonstrating that metformin reduces oxidative DNA damage in experimental models of diabetes and oxidative stress [23,27]. Conversely, novel miR-1133 mimic transfection was associated with increased 8-OHdG levels despite continued metformin treatment, suggesting that elevated novel miR-1133 expression is accompanied by increased oxidative DNA damage. Given the close relationship between oxidative DNA damage, endothelial dysfunction, and the development of diabetic vascular complications, these observations further support a potential role for novel miR-1133 in modulating endothelial oxidative stress responses.

The changes in oxidative stress observed in the present study may also be related to the accompanying alterations in PIK3CA expression. Previous studies have demonstrated that activation of PI3K signaling promotes antioxidant defense through downstream activation of Akt and subsequent nuclear factor erythroid 2-related factor 2 (NRF2)-mediated transcription of antioxidant enzymes, including SOD1 [17]. Therefore, the reduction in PIK3CA expression observed following novel miR-1133 mimic transfection raises the possibility that impaired PI3K-dependent antioxidant signaling contributed to the observed increase in oxidative stress. However, downstream components of this pathway, including Akt phosphorylation, NRF2 activation, and downstream antioxidant target genes, were not directly evaluated in the present study. Consequently, involvement of the PI3K/Akt/NRF2 axis should be regarded as a plausible mechanistic explanation based on the existing literature rather than an experimentally confirmed signaling pathway.

A major strength of the present study is that it extends our previous transcriptomic observations by providing the first functional characterization of novel miR-1133 in hyperglycemia-induced endothelial cells. Whereas our earlier small RNA sequencing study identified novel miR-1133 as a metformin-responsive miRNA through bioinformatic analyses [13], the present study demonstrates that experimental modulation of this previously uncharacterized miRNA is associated with reciprocal changes in PIK3CA expression and multiple markers of oxidative stress. Together, these findings provide an important foundation for future mechanistic investigations into the biological role of novel miR-1133 in endothelial dysfunction and diabetic vascular disease.

However, several limitations of the present study should be acknowledged. Although our previous bioinformatic analyses identified PIK3CA as a biologically plausible downstream candidate of novel miR-1133 [12,13], direct target validation using dual-luciferase reporter assay or other target engagement approaches was not performed in this study. Consequently, the inverse relationship observed between novel miR-1133 and PIK3CA expression should be interpreted as functional evidence supporting a potential regulatory relationship rather than definitive evidence of direct post-transcriptional regulation. Likewise, downstream components of the proposed PI3K/Akt signaling pathway, including Akt phosphorylation, NRF2 activation, and downstream antioxidant targets, were not evaluated. Therefore, the proposed involvement of this pathway represents a biologically plausible mechanism supported by the existing literature rather than an experimentally confirmed signaling cascade.

Furthermore, the functional significance of novel miR-1133 was investigated using a gain-of-function approach only. Although the transfection protocol was validated using a positive control miRNA mimic, novel miR-1133 expression was not directly quantified following mimic transfection, and post-transfection cell viability was not assessed. The transfection experiment included a Lipofectamine^®^-only control but did not include a scrambled negative-control mimic, a novel miR-1133 inhibitor, a mimic-treated HG-only group, multiple mimic concentrations, or a concurrent 48-h untransfected HG + Met control. Consequently, the present findings should be interpreted as demonstrating an association between novel miR-1133 mimic transfection and attenuation of antioxidant responses during ongoing metformin treatment rather than establishing the basal, independent, or dose-dependent effects of novel miR-1133. Although all transfected groups underwent identical treatment conditions and experimental duration, transient transfection itself may have introduced nonspecific cellular responses that could not be completely excluded. Accordingly, the present findings should be interpreted as hypothesis-generating evidence supporting a potential functional association among novel miR-1133, PIK3CA expression, and the antioxidant responses associated with metformin, rather than as evidence of a direct miRNA-target interaction or an established molecular mechanism.

Finally, several limitations relating to the experimental model should be considered. An osmotic control was not included; therefore, a contribution of hyperosmolarity to the observed cellular responses cannot be completely excluded. In addition, the glucose concentration (33.3 mmol/L), metformin concentration (10 µM), and treatment duration were selected to maintain consistency with our previously established HUVEC model, in which novel miR-1133 was initially identified [12,13], rather than being derived from formal dose–response or time-course optimization. The present study also examined a single post-transfection endpoint in one endothelial cell model. Moreover, although U6 was selected as the endogenous reference based on its widespread use in miRNA RT-qPCR studies, its stability was not independently validated under the present experimental conditions. Routine mycoplasma testing on primary HUVECs was also not performed. Future studies incorporating osmotic controls, systematic optimization of treatment conditions, validation of stable endogenous reference genes, routine mycoplasma testing, additional endothelial models, in vivo studies, and time-course analyses will further strengthen understanding of the biological role of novel miR-1133.

## 4. Materials and Methods

### 4.1. HUVECs Isolation and Culture

Human umbilical vein endothelial cells (HUVECs) were isolated from human umbilical cords obtained from the Labour Room, Hospital Canselor Tuanku Muhriz (HCTM), Universiti Kebangsaan Malaysia, following written informed consent from all donors. The study protocol was approved by the Universiti Kebangsaan Malaysia Research Ethics Committee (UKM PPI/111/8/JEP-2023-246). Umbilical cords were obtained from healthy term pregnancies without known metabolic or vascular complications. A total of six independent umbilical cord donors were included in the study.

HUVECs were isolated using an enzymatic digestion method as previously described [28]. Briefly, umbilical cords were rinsed with sterile cord buffer before perfusion with 0.1% Type I collagenase solution (Worthington Biochemical Corporation, Lakewood, NJ, USA) and incubated at 37 °C for 15 min. The cell suspension was collected and centrifuged at 1200 rpm for 10 min. The resulting cell pellet was resuspended in endothelial cell medium (ScienCell Research Laboratories, Carlsbad, CA, USA) supplemented with 5% fetal bovine serum, 1% endothelial cell growth supplement, and 1% penicillin/streptomycin, and cultured in T25 tissue culture flasks at 37 °C in a humidified atmosphere containing 5% CO_2_. The culture medium was replaced every two days until confluence was achieved.

Endothelial identity was confirmed by the characteristic cobblestone morphology and positive immunocytochemical staining for CD31 and von Willebrand factor. Representative immunocytochemistry images are provided in Supplementary Figure S1. Primary HUVECs at passages 3–5 were used for all experiments without undergoing freeze–thaw cycles, and treatments were initiated when cells reached approximately 80% confluence.

### 4.2. Experimental Design

For the non-transfected experiments, HUVECs were cultured under normoglycemic conditions (5.5 mmol/L glucose), hyperglycemic conditions (HG; 33.3 mmol/L glucose), or hyperglycemic conditions supplemented with 10 μM metformin (TCI Chemical, Portland, OR, USA) (HG + Met) for 24 h. Hyperglycemic conditions were established by supplementing endothelial cell medium (ScienCell Research Laboratories, Carlsbad, CA, USA) with D-glucose (Sigma-Aldrich, St. Louis, MO, USA) to achieve a final glucose concentration of 33.3 mmol/L. Cells were treated for 24 h, after which they were harvested for downstream analyses, including novel miR-1133 expression, PIK3CA mRNA and protein expression, ROS production, SOD1 mRNA expression, SOD activity, and 8-OHdG levels.

The glucose concentration (33.3 mmol/L), metformin concentration (10 μM), and treatment duration (24 h) were selected to maintain consistency with our previous small RNA sequencing study, in which identical experimental conditions were used to identify novel miR-1133 as a metformin-responsive putative miRNA [13]. These conditions were therefore adopted in the present study to facilitate functional characterization of novel miR-1133 under the same experimental model. All experiments were performed in at least three independent biological replicates.

### 4.3. Quantification of Novel miR-1133 Expression

Novel miR-1133 expression was quantified in control, HG, and HG + Met-treated HUVECs using stem-loop reverse transcription quantitative polymerase chain reaction (stem-loop RT-qPCR). Novel miR-1133 is a putative novel miRNA that was previously identified from our small RNA sequencing dataset using the miRDeep2 algorithm following alignment with the human reference genome and exclusion of known miRNAs through comparison with miRBase [12]. The candidate miRNA was subsequently validated by stem-loop RT-qPCR in our previous study [12,13] and was selected for functional characterization in the present work. As novel miR-1133 has not yet been incorporated into public miRNA databases, custom stem-loop RT-qPCR primers were designed based on its mature sequence identified from the sequencing data.

Briefly, cDNA was synthesized from 5 ng of total RNA enriched with small RNAs using the MMLV Reverse Transcriptase 1st-Strand cDNA Synthesis Kit (Biosearch Technologies, Hoddesdon, UK) and a novel miR-1133-specific stem-loop primer (0.1 μM). Quantitative PCR was subsequently performed in a total reaction volume of 10 μL containing 3 μL of 60-fold diluted cDNA, 0.5 μM each of forward and reverse primers, and 5 μL of 2× miRCURY SYBR Green Master Mix. The primer sequences used in this study are listed in Table 1.

Amplification was performed using a CFX96™ Real-Time PCR Detection System (Bio-Rad, Hercules, CA, USA) with an initial denaturation step at 95 °C for 10 min, followed by 40 cycles of denaturation at 95 °C for 10 s, annealing at 60 °C for 30 s, and extension at 72 °C for 10 s. Melting curve analysis was performed to confirm amplification specificity. Relative expression levels of novel miR-1133 were calculated using the 2^−ΔΔCt^ method and normalized to U6 small nuclear RNA (U6), which was selected as the endogenous reference because it is widely used for miRNA RT-qPCR normalization in endothelial cell studies and is recommended by the assay manufacturer.

### 4.4. Transient Transfection of Novel miR-1133 Mimic

The transfection experiment was performed to investigate the functional significance of novel miR-1133 expression in metformin-treated hyperglycemia-induced HUVECs. HUVECs were first exposed to hyperglycemic conditions supplemented with metformin (HG + Met) for 24 h. Following the initial treatment period, cells were transfected with either Lipofectamine^®^ RNAiMAX (Thermo Fisher Scientific Inc., Waltham, MA, USA) alone (transfection control) or Lipofectamine^®^ RNAiMAX complexed with a novel miR-1133 mimic (5 nM; Thermo Fisher Scientific Inc., Waltham, MA, USA), according to the manufacturer’s instructions. Cells receiving Lipofectamine^®^ RNAiMAX alone served as the transfection control to account for any nonspecific effects associated with the transfection reagent or procedure.

Hyperglycemic conditions (33.3 mM glucose) and metformin treatment (10 μM) were maintained continuously throughout the subsequent 24-h transfection period. Cells were harvested immediately after transfection for analysis of PIK3CA mRNA and protein expression, ROS production, SOD1 mRNA expression, SOD activity, and 8-OHdG. Thus, the total experimental duration for the transfection experiment was 48 h, comprising an initial 24-h HG + Met treatment followed by a 24-h transfection period under continued HG + Met conditions (Supplementary Figure S2).

Prior to the functional experiments, the transient transfection protocol was validated using the commercially available positive control miRNA mimic hsa-miR-1 (Thermo Fisher Scientific Inc., USA). Successful delivery of a biologically active miRNA mimic was confirmed by the expected reduction in expression of its known target gene, PTK9 (Supplementary Figure S3), demonstrating successful implementation of the transfection protocol. Novel miR-1133 expression was not directly quantified following mimic transfection in the functional experiments.

### 4.5. RT-qPCR for PIK3CA and SOD1 Gene Expression

Total RNA was isolated from HUVECs using the miRNeasy Mini Kit (Qiagen, Germantown, MD, USA), and cDNA was synthesized using an M-MLV reverse transcription system (Biosearch Technologies, Hoddesdon, UK). Quantitative PCR was performed to determine the mRNA expression of PIK3CA and SOD1. PIK3CA mRNA expression was quantified using commercially available PIK3CA primers (QT00014861; Qiagen, USA), whereas custom primers for SOD1 and GAPDH were designed using Primer3 software v2.6.1 based on sequences retrieved from the NCBI GenBank database. The primer sequences were as follows: SOD1 forward, 5′-GTTGCAGTCCTCGGAACCAG-3′ and reverse, 5′-CCACACCTTCACTGGTCCAT-3′; GAPDH forward, 5′-ACAGTTGCCATGTAGACC-3′ and reverse, 5′-TTGAGCACAGGGTACTTTA-3′. GAPDH was used as the housekeeping gene for normalization. Amplification was performed using BlasTaq™ 2X qPCR MasterMix in a CFX96™ Real-Time PCR System (Bio-Rad, Hercules, CA, USA). The thermal cycling conditions consisted of initial denaturation at 95 °C for 3 min, followed by 40 cycles of denaturation at 95 °C for 15 s and annealing at 58 °C for 10 s. Relative gene expression was calculated using the 2^−ΔΔCt^ method.

### 4.6. Enzyme-Linked Immunosorbent Assay (ELISA)

PIK3CA protein and 8-OHdG levels were quantified using commercially available ELISA kits according to the manufacturer’s protocols. PIK3CA protein levels were quantified in HUVEC lysates using a human PIK3CA ELISA kit (Elabscience, Houston, TX, USA), while 8-OHdG levels were measured in cell culture supernatants using a human 8-OHdG ELISA kit (Elabscience, Houston, TX, USA). Absorbance was measured at 450 nm using a microplate reader (EnSpire^®^ Multimode Plate Reader, PerkinElmer, Shelton, CT, USA). The concentrations of PIK3CA and 8-OHdG were calculated from standard curves generated using the corresponding standards provided in each kit.

### 4.7. Determination of Intracellular ROS Levels

Intracellular ROS levels were measured using the dichlorodihydrofluorescein diacetate (DCFH-DA) assay. HUVECs were seeded in six-well plates and exposed to their respective treatments for 24 h. Cells were incubated with 25 μM DCFH-DA (Elabscience, Houston, TX, USA) at 37 °C for 45 min. Following incubation, cells were harvested, washed, and resuspended in assay buffer. Fluorescence intensity was measured using a microplate reader (EnSpire PerkinElmer, USA) at excitation and emission wavelengths of 485 and 535 nm, respectively. ROS levels were expressed as relative fluorescence units (RFU).

### 4.8. Determination of Total SOD Activity

Total SOD activity was determined using the Elabscience^®^ Total Superoxide Dismutase Activity Assay Kit (Elabscience, Houston, TX, USA) according to the manufacturer’s instructions. Following treatment, cell lysates were prepared and incubated with the assay reagents in a 96-well plate. Absorbance was measured at 450 nm using a microplate reader. SOD activity was expressed as U/mg protein.

### 4.9. Statistical Analysis

Data were analyzed using GraphPad Prism version 9.0 (GraphPad Software, Boston, MA, USA). Normality was assessed using the Shapiro–Wilk test. Results are expressed as mean ± standard error of the mean (SEM). Comparisons among control, HG, and HG + Met groups were performed using one-way analysis of variance (ANOVA) followed by Tukey’s post hoc test. Comparisons between transfected groups were analyzed using an unpaired Student’s *t*-test. The number of biological replicates (*n*) is indicated in the figure legends. A *p*-value < 0.05 was considered statistically significant.

## 5. Conclusions

In conclusion, the present study demonstrates that metformin attenuates oxidative stress while enhancing endogenous antioxidant defense in hyperglycemia-induced endothelial cells. These protective effects are associated with reduced novel miR-1133 expression and increased PIK3CA expression. Furthermore, novel miR-1133 mimic transfection during continued metformin treatment is associated with reciprocal changes in PIK3CA expression, increased oxidative stress, and reduced antioxidant responses. Collectively, these findings provide the first functional characterization of novel miR-1133 and suggest that it may represent a candidate mediator of the endothelial antioxidant responses associated with metformin under hyperglycemic conditions. Further studies are required to validate the direct molecular targets of novel miR-1133, elucidate the underlying signaling mechanisms, and determine its role in metformin-mediated vascular protection.

## Figures and Tables

**Table 1:** Primer sequences used for stem-loop RT-qPCR analysis of novel miR-1133.

Target miRNA	Primer Sequence (5′-3′)		PCR Product Size (bp)
1133	Forward	GCTGGGCGGCTTGCTGG	72
	Reverse	GTAGGATGCCGCTCTCAG	
U6	Forward	CTCGCTTCGGCAGCACA	94
	Reverse	AACGCTTCACGAATTTGCGT	
Stem-loop miR-1133	GTTGGCTCTGGTAGGATGCCGCTCTCAGGGCATCCTACCAGAGCCAAACCGAGCC		

## Data Availability

The data presented in this study are available from the corresponding author upon reasonable request.

## Supplementary Materials

The following supporting information can be downloaded at: https://www.mdpi.com/article/10.3390/ijms27177556/s1.

## Abbreviations

The following abbreviations are used in this manuscript:

8-OHdG	8-hydroxy-2′-deoxyguanosine
AKT	Protein kinase B
AMPK	Adenosine monophosphate-activated protein kinase
ANOVA	Analysis of variance
CD31	Cluster of differentiation 31
CO_2_	Carbon dioxide
cDNA	Complementary DNA
CT	Threshold cycle
DCF	Dichlorofluorescein
DCF-DA	2′,7′-dichlorodihydrofluorescein diacetate
DM	Diabetes mellitus
DNA	Deoxyribonucleic acid
ELISA	Enzyme-linked immunosorbent assay
GAPDH	Glyceraldehyde-3-phosphate dehydrogenase
HG	Hyperglycemia
HUVECs	Human umbilical vein endothelial cells
Met	Metformin
miRNA	MicroRNA
mRNA	Messenger RNA
NADPH	Nicotinamide adenine dinucleotide phosphate
NRF2	Nuclear factor erythroid 2-related factor 2
PI3K	Phosphatidylinositol 3-kinase
PIK3CA	Phosphatidylinositol-4,5-bisphosphate 3-kinase catalytic subunit alpha
PKC	Protein kinase C
qRT-PCR	Quantitative real-time polymerase chain reaction
ROS	Reactive oxygen species
SEM	Standard error of the mean
SOD	Superoxide dismutase
SOD1	Superoxide dismutase-1
