# Supplementary material for: Functional Characterization of Putative Novel MicroRNA-1133 in the Antioxidant Effects of Metformin in Hyperglycemia-Induced Endothelial Cells
Source: Int J Mol Sci. 2026 Aug 24;27(17):7556. doi: 10.3390/ijms27177556 (PMC13566550; doi:10.3390/ijms27177556)
Supplement: Supplementary file 1 [file ijms-27-07556-s001.zip › ijms-4474248-supplementary.pdf]

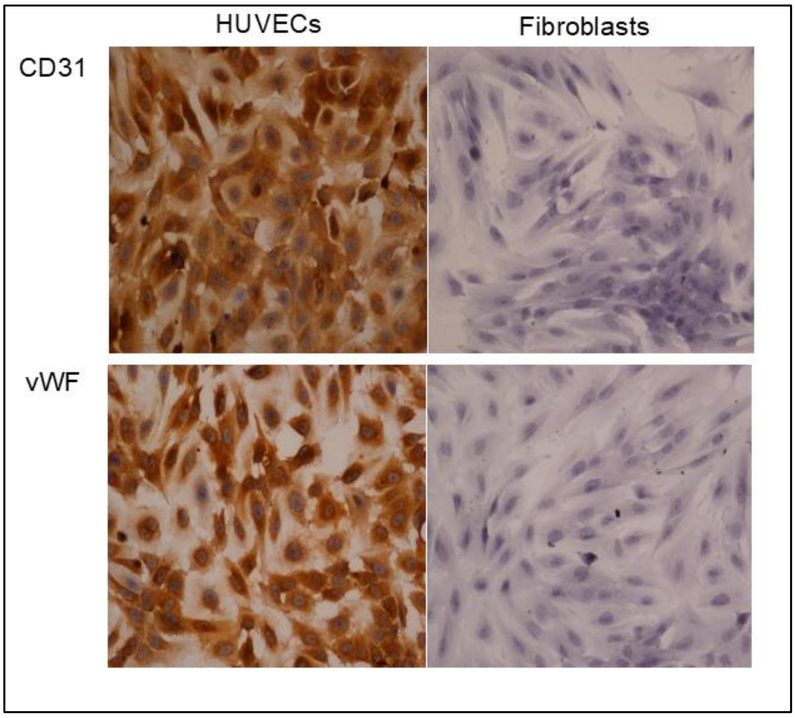

Supplementary Figure S1. Characterization of primary human umbilical vein endothelial cells (HUVECs). Representative immunocytochemical staining of HUVECs and fibroblasts (negative control) for the endothelial markers CD31 and von Willebrand factor (vWF) at ×200 magnification. HUVECs demonstrated strong positive immunostaining for both CD31 and vWF, whereas fibroblasts showed negative staining, confirming the endothelial identity of the isolated primary HUVECs.

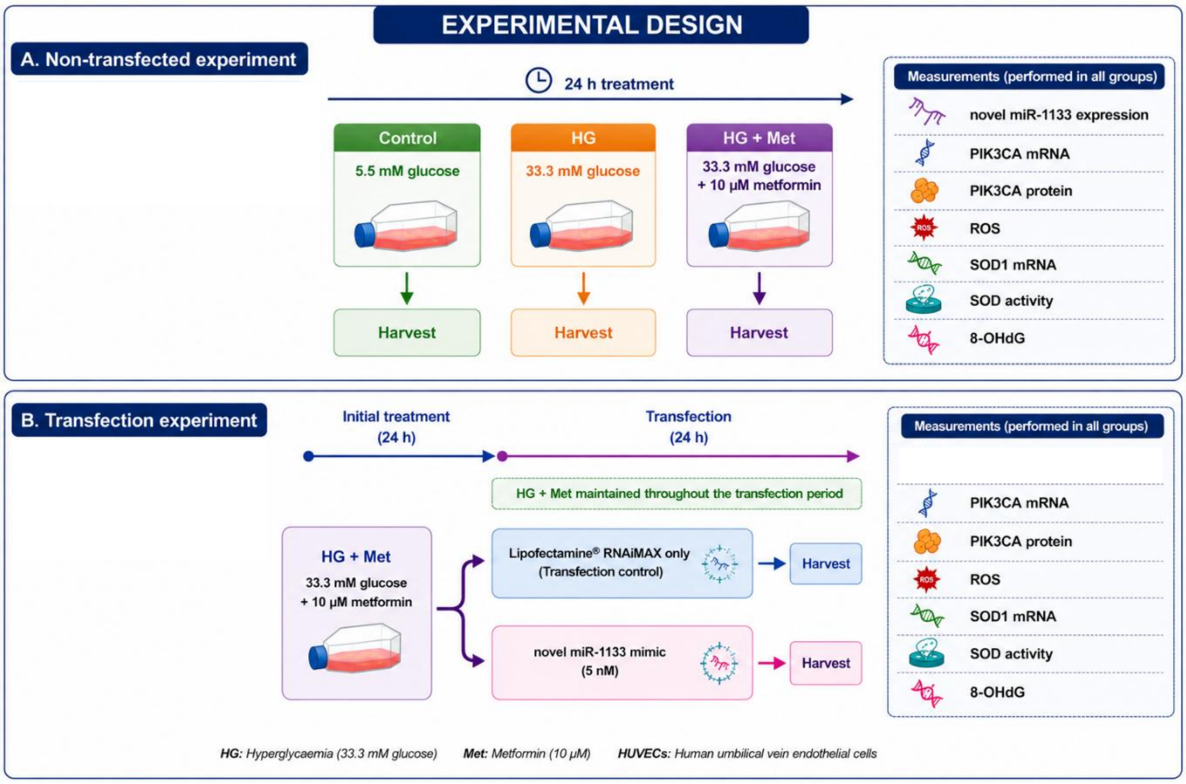

Supplementary Figure S2. Schematic illustration of the experimental design. **(A)** Non-transfected experiment: HUVECs were cultured under normoglycemic conditions (5.5 mmol/L glucose), hyperglycemic conditions (HG; 33.3 mmol/L glucose), or hyperglycemic conditions supplemented with metformin (HG+Met; 10 μM) for 24 h before analysis of novel miR-1133 expression, PIK3CA mRNA and protein expression, intracellular reactive oxygen species (ROS), SOD1 mRNA expression, total superoxide dismutase (SOD) activity, and 8-hydroxy-2′-deoxyguanosine (8-OHdG) levels. **(B)** Transfection experiment: HUVECs were initially treated with HG+Met for 24 h, followed by transfection with either Lipofectamine® RNAiMAX alone (transfection control) or a novel miR-1133 mimic (5 nM) during continued HG+Met treatment for an additional 24 h before downstream analyses.

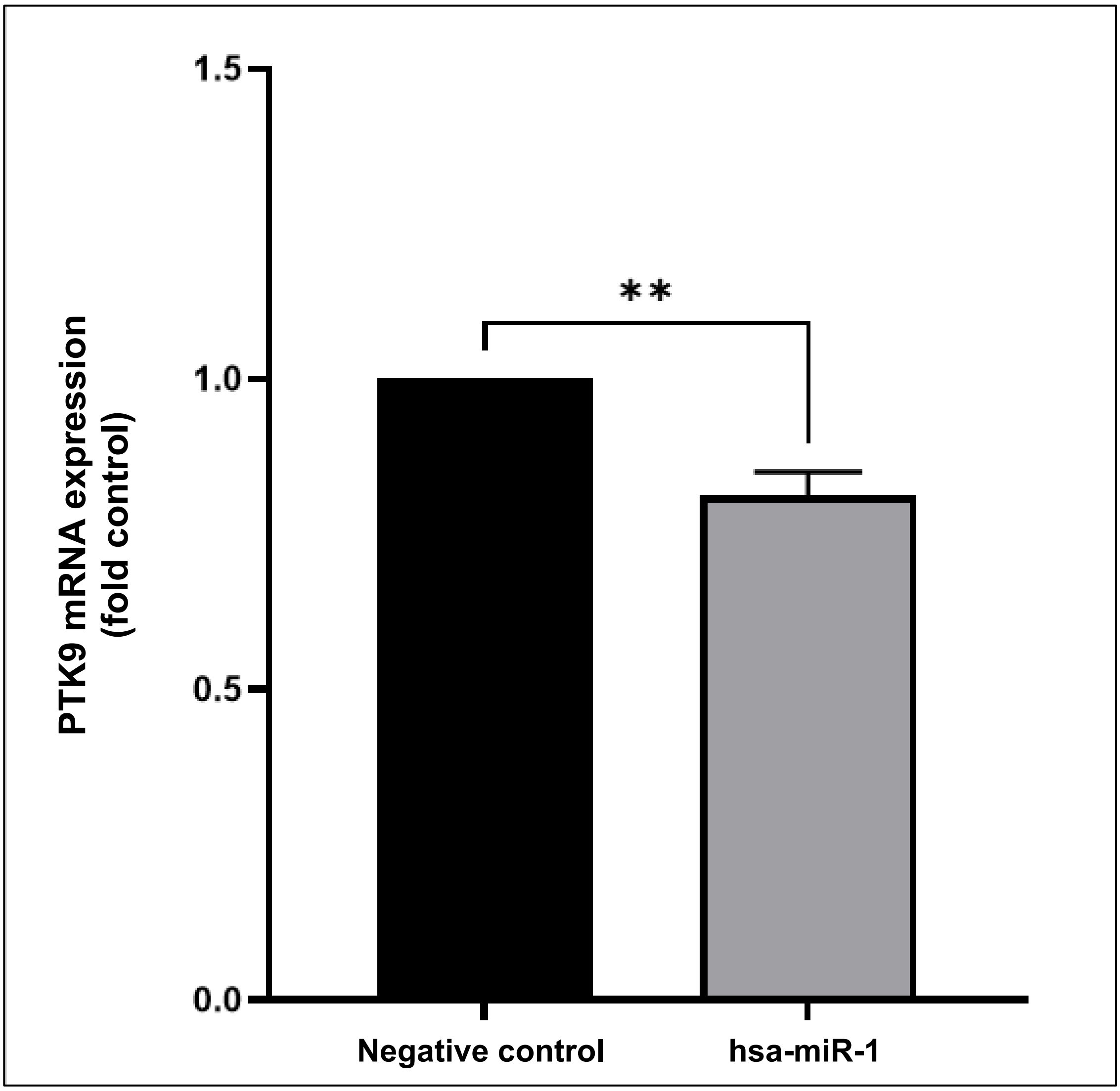

Supplementary Figure S3. Validation of the transient transfection protocol using the positive control miRNA mimic hsa-miR-1. HUVECs were transfected with the commercially available hsa-miR-1 mimic, and successful implementation of the transfection protocol was confirmed by the expected reduction in expression of its known target gene, PTK9, compared with the corresponding transfection control. This experiment validated the transient transfection protocol used in the functional studies. Data are presented as mean ± SEM, n = 3, ** $p < 0.01$.
